# Dysregulated Arginine Metabolism in Young Patients with Chronic Persistent Asthma and in Human Bronchial Epithelial Cells

**DOI:** 10.3390/nu13114116

**Published:** 2021-11-17

**Authors:** Bo Zhou, Gulinigaer Tuerhong Jiang, Hui Liu, Manyun Guo, Junhui Liu, Jianqing She

**Affiliations:** 1 Respirotory Department, The First Affiliated Hospital of Xi’an Jiaotong University, No. 277 W. Yanta Road, Xi’an 710061, China; zb_bob@stu.xjtu.edu.cn; 2 Cardiovascular Department, The First Affiliated Hospital of Xi’an Jiaotong University, No. 277 W. Yanta Road, Xi’an 710061, China; gulinigar@stu.xjtu.edu.cn (G.T.J.); gmylucyele@stu.xjtu.edu.cn (M.G.); 3 Biobank, First Affiliated Hospital of Xi’an Jiaotong University, Xi’an 710061, China; liuh2018@xjtu.edu.cn; 4 Diagnostic Department, The First Affiliated Hospital of Xi’an Jiaotong University, No. 277 W. Yanta Road, Xi’an 710061, China; liu1109@xjtu.edu.cn

**Keywords:** chronic persistent asthma, human airway epithelial cells, arginine metabolism, L-citrulline

## Abstract

Background: Recent metabolomics studies have found circulatory metabolism alterations in patients with asthma, indicating that altered metabolites played a significant role in asthma. However, the regulatory mechanisms in asthma, especially in young chronic persistent asthma remain underexplored. Methods: In this study, a prospective cohort of 162 patients diagnosed of asthma admitted to the First Affiliated Hospital of Xi’an Jiaotong University from January 2018 to December 2019 was used to perform a nested case-control study. Among them, we included 30 patients with chronic persistent asthma between 20 to 35 years old; 30 health control with evenly distributed age and sex were then recruited. Nontargeted metabolomics was applied to identify serum metabolic profiles and altered metabolic pathways. Results: In vitro, human bronchial epithelial cells (HBECs) line BEAS-2B with the addition of L-citrulline and/or asymmetric dimethylarginine (ADMA) model was utilized and the concentrations of nitric oxide (NO) metabolites were tested to evaluate the therapeutic potential of L-citrulline. The young patients with chronic persistent asthma displayed dysregulated serum metabolic profiles, especially enriched in arginine metabolism. The ratio of L-citrulline to ornithine is associated with blood eosinophil count. In vitro, adding L-citrulline could reverse ADMA-mediated reduction of NOx at lower L-arginine concentration (25 μM), but was ineffective in the higher L-arginine concentration (100 μM) media. Conclusions: The arginine metabolism balance is of vital importance during the pathogenesis and progression of chronic asthma. L-citrulline could be a powerful approach to restore airway NO production, potentially exhibiting therapeutic benefits among young patients with chronic asthma.

## 1. Introduction

Bronchial asthma, as a kind of chronic inflammatory diseases, is characterized by reversible airway hyperresponsiveness (AHR) and chronic airway remodeling. Allergen inhalation results in inflammatory producing factors such as IL-4, IL-5, IL-13 and TNFα in Th2 and mast cells [1]. Then, eosinophils are increased in blood and bronchoalveolar lavage fluid (BALF). In the course, the airway epithelium is thought to play an important role in the pathogenesis of asthma. Activated stress of airway epithelial cells may contribute to inflammation and airway remodeling in the development of asthma [2]. However, the regulatory mechanisms in asthma, especially in young chronic persistent asthma remain underexplored.

Recent metabolomics studies have found circulatory metabolism alterations in some patients with asthma, indicating that L-citrulline metabolism plays a significant part in children with asthma and was considered as one important biomarker [3,4]. In healthy adults, the biosynthesis of endogenous L-arginine from L-citrulline is sufficient, with arginase Ι as an enzyme catalyzing arginine to produce ornithine [5]; however, this metabolic process is disturbed in asthma patients with decreased arginine production. Moreover, L-arginine is further found crucial for endogenous biosynthesis of nitric oxide (NO), a bronchodilator and a mediator of inflammation, preventing the progression of asthma. However, the downstream effect of altered metabolite profiles related to Arginine pathway is complex; especially, there are still limited evidence as to the altered metabolic profile and pathways in young adults with chronic persistent asthma.

In this study, we investigated in vivo the metabolite profiles in the serum of young patients with chronic persistent asthma, aiming to detect the key altered serum metabolites and correlated metabolic pathways. We have identified significant alteration of L-citrulline and L-arginine and its ratio to ornithine levels, correlating to the eosinophilia count. Moreover, in vitro, we used human bronchial epithelial cells (HBECs) line BEAS-2B and investigate the mechanism how L-citrulline restore NO production stimulated by high or low level of arginine. The aim of this study was to reveal the key function of arginine metabolism among young patients with chronic persistent asthma and the potential therapeutic value of the amino acids for asthma.

## 2. Materials and Methods

### 2.1. Human Participants and Laboratory Tests

In this study, a prospective cohort of 162 patients diagnosed of asthma admitted to the First Affiliated Hospital of Xi’an Jiaotong University from January 2018 to December 2019 was used to perform a nested case-control study. Among them, we included 30 patients with chronic persistent asthma between 20 to 35 years old; 30 healthy participants with evenly distributed age and sex recruited from the data of physical examination center in the same hospital during the same period were then recruited. Patients with arginine/citrulline metabolism disorders or arginine deficiency were excluded. Chronic persistent asthma. referring to the intermittent symptoms of chest tightness, shortness of breath and cough, which do not reach the diagnosis of acute attack period and clinical remission period, was diagnosed according to the Global Initiative for Asthma [6] and the conventional inhalation therapy was given according to the present guidelines. Generally, 160 μg Budesonide/4.5 μg Formoterol inhalation therapy (twice a day) was given for the patients for the control of chronic persistent asthma. The subjects were excluded if they were taking or had previously taken any drugs known to influence lipid metabolism or the endocrine system; had chronic cardiac disease or endocrine disease; were undergoing hemodialysis for renal failure; had acute or chronic hepatitis with increased transaminase activities; or had malignant tumor.

Demographic information was obtained as previously described [7,8,9]. Biochemical measurement, including the complete blood count with serum eosinophilia count were measured after overnight fast and sample collection. Asthma control test (ACT) was conducted according to the contemporary guidelines [10]. Written informed consent was obtained according to the Declaration of Helsinki, and was approved by the ethics committee, Xi’an Jiaotong University (NO. 2018-G169).

### 2.2. Serum Sample Preparation and Metabolism Profile Determination

Serum samples were collected from the asthma and control subjects upon enrollment after overnight fast. Venous blood was centrifuged afterwards at 3000 rpm for 10 min at 4 °C. Serum was separated and stored at −80 °C and aliquots were thawed for further processing as previously described [7,9]. The untargeted metabolomics profiling was performed on XploreMET platform (Metabo-profileTM, Shanghai, China) [7,9]. The sample preparation, instrumentation, metabolic annotation and data analysis procedure were referred in their previously published methods with minor modification [11,12,13].

### 2.3. Cell Culture

HBECs line BEAS-2B were purchased from the American Type Culture Collection (ATCC, USA), and cultured in DMEM/F12 medium with 10% fetal bovine serum (FBS). Moreover, the experiments were, respectively, performed with 100 and 25 μM of L-arginine containing media. The medium was replaced by a fresh medium prior to each experiment. When the epithelial cells reached 70% to 80% confluence, they were dissociated with trypsin-EDTA and passed onto collagen-coated polyester trans-well inserts of 12 mm in diameter (pore size, 0.4 μm) at 4 × 109 cells/cm^2^. After a week of being in immersed culture, epithelial cells reached 100% confluence and were shifted to an air–liquid interface (ALI) condition by removing all but 50 μL of the apical medium. Cell media were changed every other day. After 8 days in ALI state, cells are treated with 100 μM ADMA. To determine the effect of L-citrulline on NOx production, 1600 μM L-citrulline was added at day 9th. After 10 days, cells are harvested and media collected for analysis.

### 2.4. NOx (Nitrites + Nitrates) Determination

Lower supernatant nitrite and nitrate concentration were quantified using a colorimetric assay based on the Griess reaction (Parameter Total Nitric Oxide and Nitrite/Nitrate Assay; R&D System Inc., 614 McKinley Pl, Minneapolis, MN, USA). Briefly, nitrite was quantified and nitrate was converted to nitrite using nitrate reductase, followed by the addition of Griess reagent to produce an azo dye compound. The absorbance was measured at 540 nm. NOx concentration was calculated from a standard curve prepared from serial nitrite dilution. The experiments were repeated 3 times.

### 2.5. Statistical Analysis

Data were presented as mean ± SE. for continuous variables and percentage for categorical variables. The correlation analysis between significantly altered metabolites and serum eosinophilia counts was performed using Pearson’s correlation (SPSS 20.0). Enrichment analyses were performed based on pathway-associated metabolite sets [14,15]. The volcano plot and enrichment pathway plot were created using R studio and Prism 9.0. *p*-values < 0.05 were considered as significant.

## 3. Results

### 3.1. Clinical Characteristics of the Subjects Enrolled

**Figure 1:**
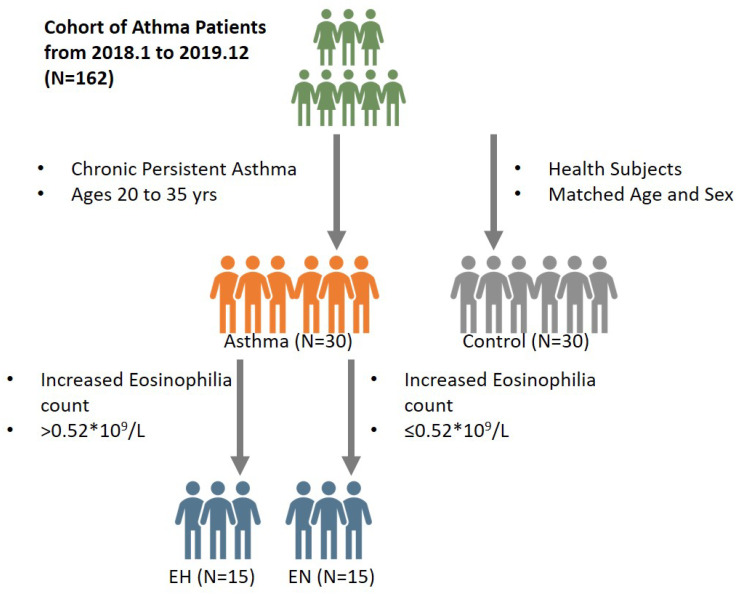
Cohort enrollment.

This is a single-center nested-cohort study enrolled a total of 162 patients diagnosed of asthma admitted to the First Affiliated Hospital of Xi’an Jiaotong University from January 2018 to December 2019. Among them, we included 30 patients with chronic persistent asthma between 20 to 35 years old; 30 health control with evenly distributed age and sex were then selected. 15 asthma patients had blood eosinophilia count within normal range and 15 with higher levels of eosinophilia count (Figure 1). We evaluated serum metabolic profile of 30 healthy and 30 chronic persistent asthma adults aging from 20 to 35 years old. Baseline information of participants enrolled was shown in Table 1. No significant difference in age, gender and body mass index (BMI) were seen between chronic persistent asthma patients and healthy control. Of note, eosinophil count was increased in the patients with chronic persistent asthma compared to control, and ACT was 22.5 ± 2.1 points in the patients with chronic persistent asthma.

### 3.2. Comprehensive Metabolomics Profile and Composition in Asthma Patients and Control

**Figure 2:**
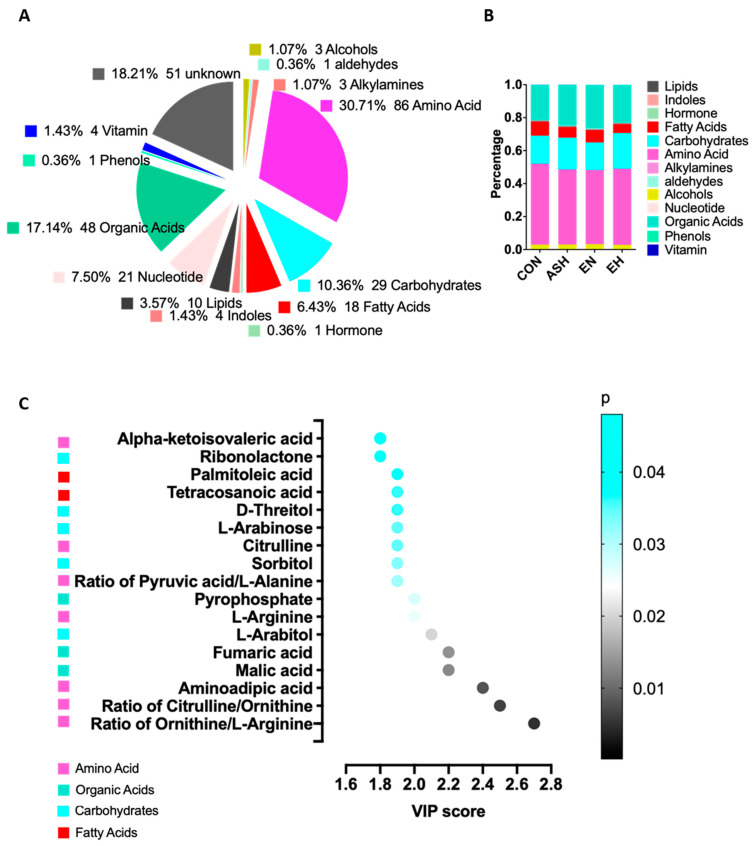
Circulating metabolomics profile and composition in asthma and control cohorts. (**A**) The serum metabolite kinds detected by the untargeted metabolomics profiling. (**B**) The compositions of each metabolite kinds indicated by respective percentage. (**C**) Variable importance in projection (VIP) and correlation coefficients assessment of significant metabolites and ratio between control and asthma calculated using an OPLS-DA model.

To further investigate metabolic profile alteration in the chronic persistent asthma, the untargeted metabolomics profiling was performed and a total of 280 metabolites were detected, including amino acids, organic acids, carbohydrates, nucleotides, and so on (Figure 2A). The compositions of each metabolite kinds indicated by respective percentage were generally the same between control, asthma, asthma with high eosinophilia and asthma with normal eosinophilia groups (Figure 2B). Variable importance in projection (VIP) and correlation coefficients assessment of significant metabolites and ratio between control and asthma was calculated using an OPLS-DA model (Figure 2C, Supplementary Table S1). The most significantly altered metabolites and its ratio and classes were ratio of ornithine/L-arginine, ratio of citrulline/ornithine, aminoadipic acid, L-arginine, citrulline, ratio of pyruvic acid/L-alanine, and alpha-ketoisovaleric acid belonging to amino acids, malic acid, fumaric acid and pyrophosphate belonging to organic acids, L-arabitol, sorbitol, L-arabinose, D-threitol, ribonolactone belong to carbohydrates, and tetracosanoic acid and palmitoleic acid belonging to fatty acids.

### 3.3. Significantly Altered Pathways during Chronic Persistent Asthma

**Figure 3:**
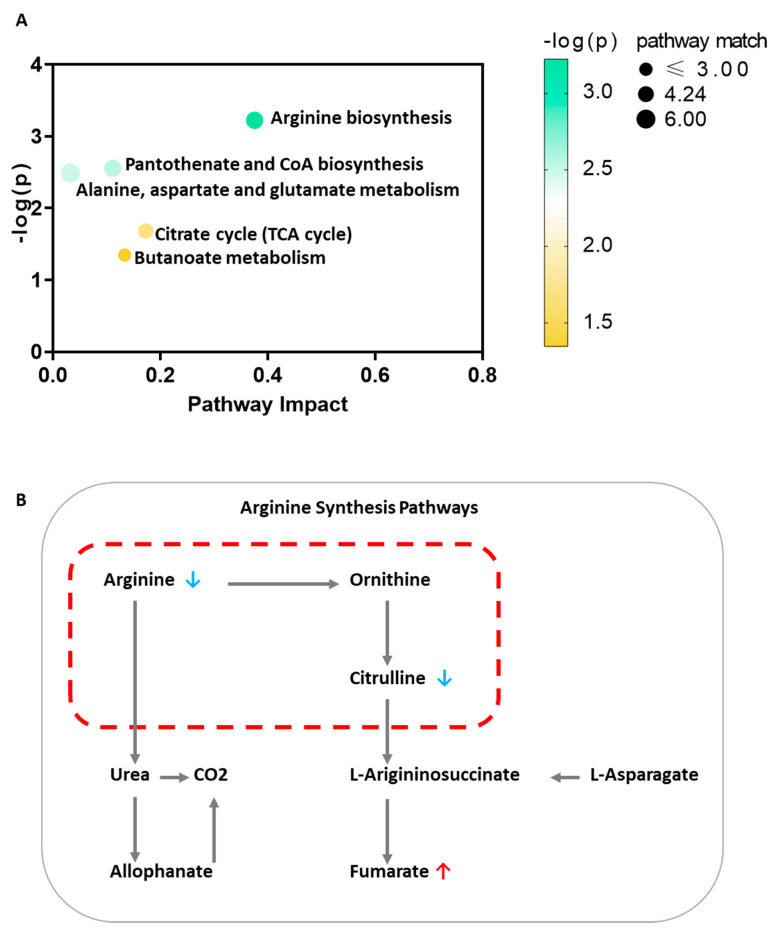
Significantly altered pathways during chronic persistent asthma. (**A**) Significantly altered pathways during chronic persistent asthma. X axis indicated the pathway impact and Y axis indicated minus log transformed *p* value. The node size of each pathway indicated the pathway match. (**B**) Arginine synthesis pathways indicating decreased Arginine and Citrulline, and increased Fumarate.

To further investigate disturbed metabolic pathways in chronic persistent asthma patients, we performed the pathway analysis to identify significantly altered pathways based on metabolites involvement in the same biological pathways. In line with metabolites identification, the arginine biosynthesis pathway was found mostly differentially altered (Figure 3A) with down regulation of L-arginine and citrulline, and up regulation of Citrulline (Figure 3B).

### 3.4. Significantly Dysregulated Amino Acids and Their Ratios in Asthma and Control Cohort

**Figure 4:**
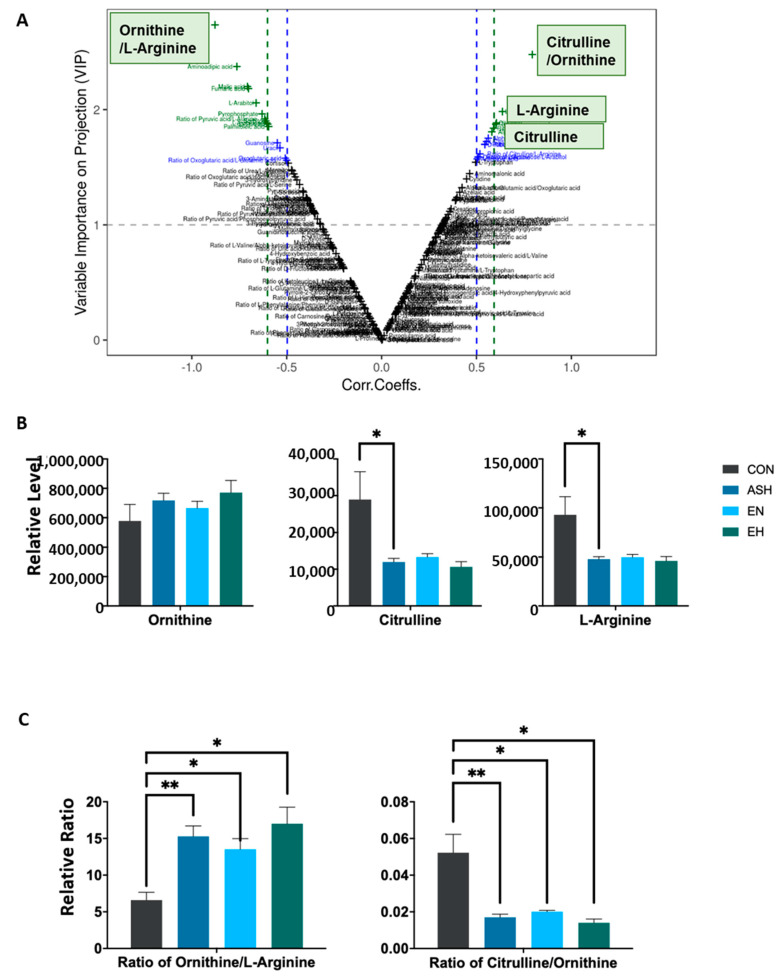
Significantly dysregulated amino acids and their ratios in asthma and control cohorts. (**A**) The volcano-plot combining the strength of both contribution (variable importance in projection) and variable reliability (correlation coefficients). The value of VIP score which is greater than 1 is the typical rule for selecting relevant variables. With a significance level of 0.05, a corresponding Corr. value is used as a cutoff value to select the variables that are most correlated with the very first predictive component. The green metabolites were reliable metabolite markers based on VIP and Corr. Of interest, the metabolites L-arginine and citrulline and the metabolites ratio of ornithine/L-arginine and citrulline/ornithine exhibited VIP score greater than 1 with significance corresponding Corr. (**B**) Relative serum levels of L-arginine, citrulline and ornithine and the ratio of ornithine/L-arginine and citrulline/ornithine (**C**) among asthma, asthma with normal eosinophilia, asthma with high eosinophilia and control participants. Data were analyzed using the one-way ANOVA. Mean ± s.e.m. * *p* < 0.05. ** *p* < 0.01.

Since the enrichment analysis points to the mostly differentially regulated arginine metabolism pathway. We carried on to evaluate the differential associated metabolites. The volcano-plot combining the strength of both contribution (VIP) and variable reliability (correlation coefficients, Corr.) has been proposed for reliable metabolite marker selection. Of note, both the metabolites L-arginine and citrulline and the metabolites ratio of Ornithine/L-arginine and citrulline/Ornithine exhibited VIP score greater than 1 with significance corresponding Corr. Values, indicating these metabolites and ratios were reliable metabolite markers for asthma (Figure 4A). We next compared the relative levels of L-arginine, citrulline and ornithine among asthma, asthma with normal eosinophilia, asthma with high eosinophilia and control participants. L-arginine and citrulline, but not ornithine, were significantly lower among asthma patients (Figure 4B). Interestingly, the ratio of ornithine/L-arginine is significantly lower, and ratio of citrulline/ornithine significantly higher in the control as compared to asthma with normal or high eosinophilia count (Figure 4C).

### 3.5. Coefficients between Significantly Altered Metabolites and Serum Eosinophilia Counts

From the metabolites investigation, the altered arginine biosynthesis pathway along with down regulation of L-arginine and citrulline were identified in young chronic persistent asthma patients. We therefore asked the question whether those metabolites were correlated to disease activity during asthma. To this end, we performed correlation analysis between all the significantly altered metabolites and blood eosinophilia counts (Table 2). The ratio of citrulline/ornithine was found significantly negatively correlated to eosinophilia counts, indicating potential interrelation between citrulline metabolism and asthma severity.

### 3.6. Addition of L-Citrulline Prevented Asymmetric Dimethylarginine (ADMA)-Mediated Reduction of NO Metabolites in HBECs

**Figure 5:**
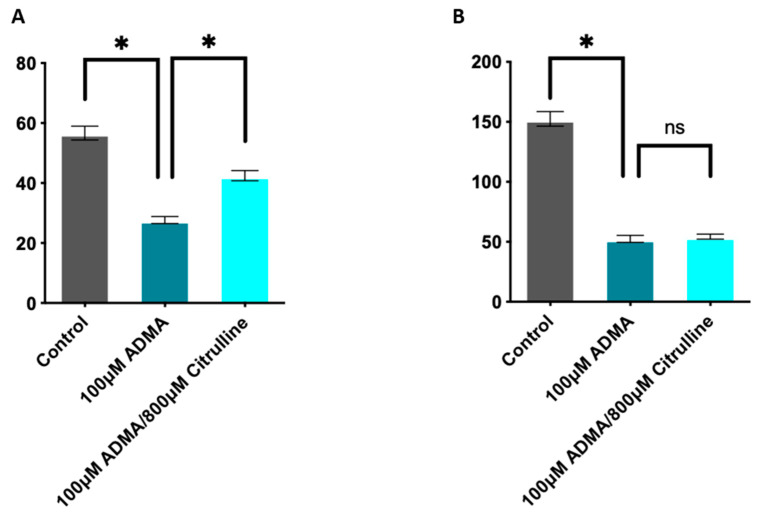
Addition of L-citrulline prevented asymmetric dimethylarginine (ADMA)-mediated reduction of NO metabolites in HBECs. (**A**). NO metabolites measurement in human bronchial epithelial cells (HBECs) exposed to low (25 μM) L-arginine cell media concentration; (**B**). NO metabolites measurement in HBECs exposed to high (100 μM) L-arginine cell media concentration. (*n* = 3–5 repeats per group). All experiments were repeated 3 times. Mean ± s.e.m. ns not significant. * *p* < 0.05.

The cohort metabolomics data showed the reduced systemic levels of L-arginine and L-citrulline in young patients with chronic persistent asthma, but how this imbalance leads to reduced nitric oxide (NO) formation in asthma and greater oxidative stress is unknown. We then cultured human bronchial epithelial cells (HBECs) line BEAS-2B and found that the addition of 100 μM of ADMA significantly decreased NOx concentration in HBECs, but L-citrulline could reverse ADMA-mediated reduction in NOx at lower (25 μM) L-arginine concentration in the media (Figure 5A), correlating to the previous study [16]. However, L-citrulline was ineffective at higher (100 μM) L-arginine concentration in the media (Figure 5B) indicating that HBECs could synthesize L-arginine from L-citrulline under specified conditions.

## 4. Discussion

In our study, we firstly focused on the Arginine pathway and their associated metabolites in young patients with chronic persistent asthma. Our results showed that the young patients with chronic persistent asthma displayed dysregulated serum metabolic profiles, especially enriched in arginine metabolism. L-citrulline and L-arginine concentrations were significantly lowered in chronic persistent asthma participants. The ratio of L-citrulline to ornithine is significantly negatively associated with blood eosinophil count. In vitro, adding L-citrulline could reverse ADMA-mediated reduction of NOx at lower L-arginine concentration (25 μM), but was ineffective in the higher L-arginine concentration (100 μM) media. The arginine metabolism balance is of vital importance during the pathogenesis and progression of chronic asthma. 

The major novelty of the present study is that we have identified the benefit of L-citrulline in asthma. Previous studies provided clues that L-citrulline metabolism dysregulation contributed to pathogenesis of asthma in children [17]. Supplemental citrulline may aid NO synthesis, which work in various ways to oppose pathogenic mechanisms in asthma [18]. Observational studies also noted that the biosynthesis of endogenous L-arginine from L-citrulline is disturbed in asthma patients [19]. Although there is emerging evidence about the protective functions of L-citrulline during asthma, few clinical studies have evaluated the comprehensive metabolic profile and associated pathways in young chronic persistent asthma adults.

The particularly significant role of L-arginine in bronchial asthma lies in its involvement in biosynthesis of polyamines and NO [20], which is produced by a family of isoenzymes and all utilize L-arginine as substrate [19]. Thus, decreased level of L-citrulline and L-arginine may lead to airway hyper responsiveness, inflammation, and airway remodeling in the development of asthma. In our study, it was identified that in vivo, serum L-citrulline and L-arginine levels were significantly reduced in asthma patients. In vitro, it was further investigated that L-citrulline could restore NO production in human bronchial epithelial cells. As we known, the production of NO could be altered by increased competition between NOS and the arginase pathway, which produces urea and ornithine. It have proved that the airways hypercontractility in animal models of asthma was in part related to imbalances in the production of NO by the neuronal NOS, as well as increased competition for substrate L-arginine with the arginases [21]. The production of the endogenous NOS inhibitor, asymmetric dimethylarginine (ADMA), as well as accumulation of the L-ornithine–derived polyamines, downstream of the arginase pathway, may also modify NO production and airway tone in asthma [22]. Additionally, as arginase is upregulated in asthma, increase L-arginine concentration may ultimately result in increased arginase activity and the formation of polyamines and proline. The polyamine spermine was recently shown to contribute to airway hyperresponsiveness in asthma by inhibiting NOS, and proline may contribute to airway remodeling, because it is a precursor of collagen formation [23]. Therefore, arginase inhibition may be a more elegant approach than L-arginine or L-citrulline supplementation.

The present results also provide evidence for benefits of L-citrulline in asthma, providing further evidence of targeting therapeutic strategies in the L-citrulline metabolic pathway. Recent studies have shown that supplementation with L-arginine increase FeNO, and reduce airway inflammation and bronchial hyperresponsiveness in murine asthma models [24]. Moreover, L-arginine supplementation inhibits oxidative stress in HBECs [25]. Various therapeutic strategies to increase L-arginine levels or bioavailability, including supplementation of L-arginine or L-citrulline and inhibition of arginase, are currently being explored [19]. However, evidence application of L-arginine supplemental therapy is limited, as L-arginine metabolism firstly occurred in the liver and intestine. Whereas, L-citrulline has been identified plausible as an amino acid supplement therapy [17]. In the present study, we focused on young patients with chronic persistent asthma. Our results further showed that L-citrulline supplementation increases L-arginine concentration and NO, further suggesting that L-citrulline supplementation could be an effective treatment for asthma.

Some limitations must be considered in this study. First, our study was a relatively small sample cohort focusing on young patients with chronic persistent asthma. As a result, the present results could not be generalizable in all asthma patients. Second, in vitro our results in HBECs could not completely reflect the changes of L-citrulline in vivo, which should be in-depth explored in the future. Third, the role of L-citrulline in vivo and its therapeutic potential require further studies. At last, because the present cohort followed up patients from the outpatient department, only the blood routine test was rechecked during the follow-up, and other values such as IgE were not available.

## 5. Conclusions

In conclusion, the present results have identified decreased levels of L-citrulline and L-arginine in young patients with chronic persistent asthma. The ratio of L-citrulline to ornithine is significantly negatively associated with blood eosinophil count. The arginine metabolism balance is of vital importance during the pathogenesis and progression of chronic asthma. L-citrulline could be a powerful approach to restore airway NO production, potentially exhibiting therapeutic benefits among young patients with chronic asthma.

## Figures and Tables

**Table 1:** Clinical characteristics of the patients with asthma and control.

Characteristics	Asthma (n = 30)	Control (n = 30)	*p*
Age (years)	25.5 ± 2.3	25.0 ± 2.8	ns
Female (%)	50%	50%	ns
BMI (kg/m^2^)	21.4 ± 2.7	20.8 ± 2.3	ns
Eosinophil count (*10^9^/L)	0.35 ± 0.12	0.16 ± 0.08	<0.01
ACT (points)	22.5 ± 2.1	-	-

ACT: asthma control test, BMI body mass index.

**Table 2:** Coefficients between significantly altered metabolites and serum eosinophilia counts.

Class	Name	Corr.	*p*
Amino Acid	Ratio of Citrulline/Ornithine	−0.84	0.04
Carbohydrates	L-Arabitol	0.84	0.04
Fatty Acids	Palmitoleic acid	−0.80	0.06
Amino Acid	Ratio of Ornithine/L-arginine	0.71	0.11
Amino Acid	Citrulline	−0.66	0.15
Carbohydrates	Sorbitol	0.65	0.16
Amino Acid	L-arginine	−0.63	0.18
Carbohydrates	L-Arabinose	0.52	0.29
Organic Acids	Malic acid	−0.58	0.30
Fatty Acids	Tetracosanoic acid	−0.48	0.33
Carbohydrates	Ribonolactone	0.44	0.38
Carbohydrates	D-Threitol	0.39	0.44
Organic Acids	Pyrophosphate	−0.35	0.49
Amino Acid	Aminoadipic acid	−0.33	0.53
Amino Acid	Ratio of Pyruvic acid/L-Alanine	−0.16	0.76
Amino Acid	Alpha-ketoisovaleric acid	0.08	0.88
Organic Acids	Fumaric acid	0.03	0.96

## Data Availability

The datasets used and/or analyzed during the current study are available from the corresponding author on reasonable request.

## Supplementary Materials

The following are available online at https://www.mdpi.com/article/10.3390/nu13114116/s1, Table S1: Variable importance in projection and correlation coefficients assessment of significant metabolites and ratio between control and asthma in an OPLS-DA model.

## Abbreviations

ACT	Asthma Control Test
ADMA	Asymmetric Dimethylarginine
AHR	Airway Hyperresponsiveness
BALF	Bronchoalveolar Lavage Fluid
BMI	Body Mass Index
HBECs	Human Bronchial Epithelial Cells
NO	Nitric Oxide
