# Supplementary material for: Dysregulated Arginine Metabolism in Young Patients with Chronic Persistent Asthma and in Human Bronchial Epithelial Cells
Source: Nutrients. 2021 Nov 17;13(11):4116. doi: 10.3390/nu13114116 (PMC8622016; doi:10.3390/nu13114116)
Supplement: Supplementary file 1 [file nutrients-13-04116-s001.zip › nutrients-1394716-supplementary.pptx]

## Slide 1
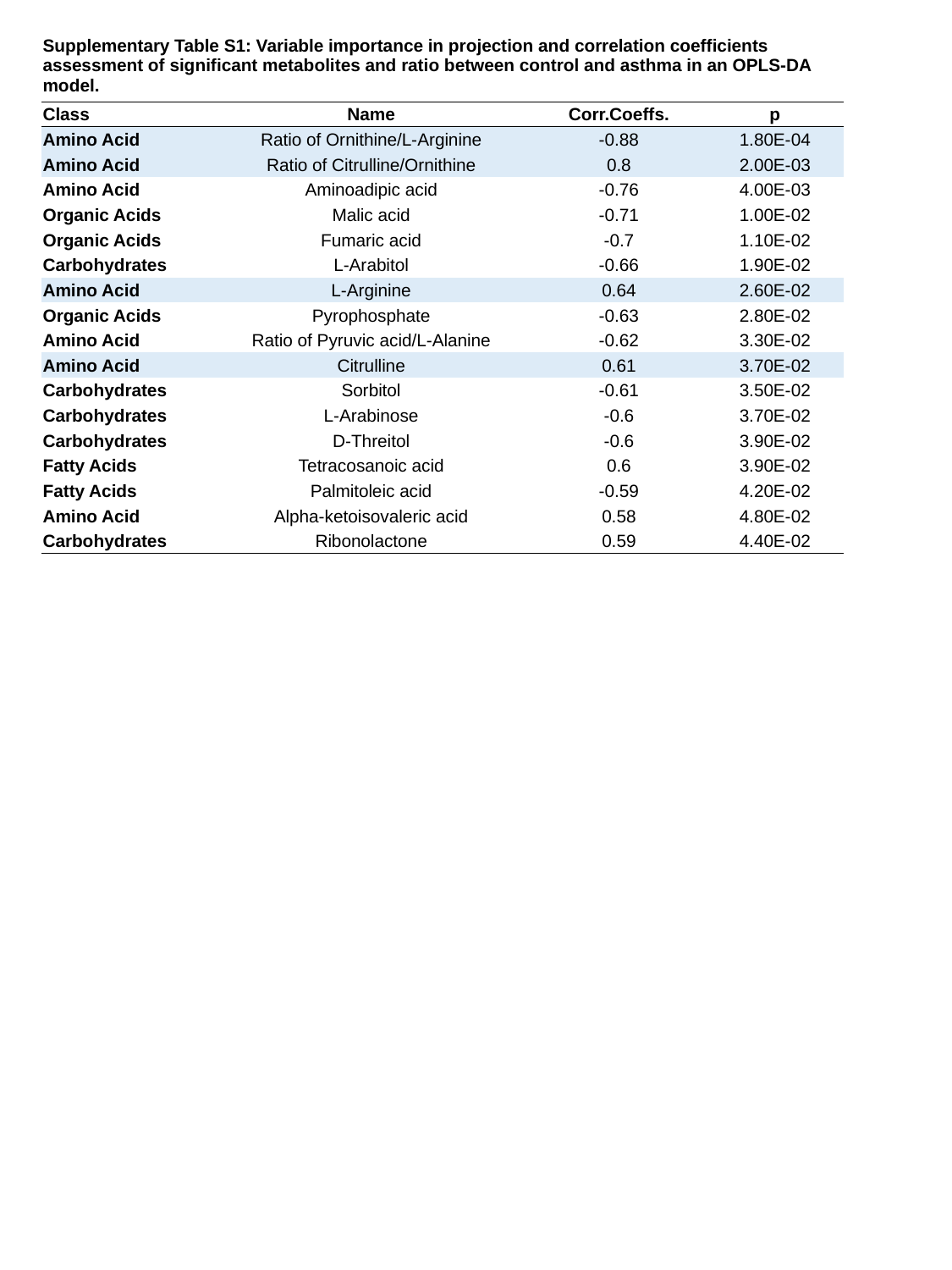

| Supplementary Table S1: Variable importance in projection and correlation coefficients assessment of significant metabolites and ratio between control and asthma in an OPLS-DA model. | | | |
| --- | --- | --- | --- |
| Class | Name | Corr.Coeffs. | p |
| Amino Acid | Ratio of Ornithine/L-Arginine | -0.88 | 1.80E-04 |
| Amino Acid | Ratio of Citrulline/Ornithine | 0.8 | 2.00E-03 |
| Amino Acid | Aminoadipic acid | -0.76 | 4.00E-03 |
| Organic Acids | Malic acid | -0.71 | 1.00E-02 |
| Organic Acids | Fumaric acid | -0.7 | 1.10E-02 |
| Carbohydrates | L-Arabitol | -0.66 | 1.90E-02 |
| Amino Acid | L-Arginine | 0.64 | 2.60E-02 |
| Organic Acids | Pyrophosphate | -0.63 | 2.80E-02 |
| Amino Acid | Ratio of Pyruvic acid/L-Alanine | -0.62 | 3.30E-02 |
| Amino Acid | Citrulline | 0.61 | 3.70E-02 |
| Carbohydrates | Sorbitol | -0.61 | 3.50E-02 |
| Carbohydrates | L-Arabinose | -0.6 | 3.70E-02 |
| Carbohydrates | D-Threitol | -0.6 | 3.90E-02 |
| Fatty Acids | Tetracosanoic acid | 0.6 | 3.90E-02 |
| Fatty Acids | Palmitoleic acid | -0.59 | 4.20E-02 |
| Amino Acid | Alpha-ketoisovaleric acid | 0.58 | 4.80E-02 |
| Carbohydrates | Ribonolactone | 0.59 | 4.40E-02 |
